# The transcription factor PHR1 regulates lipid remodeling and triacylglycerol accumulation in *Arabidopsis thaliana* during phosphorus starvation

**DOI:** 10.1093/jxb/eru535

**Published:** 2015-02-13

**Authors:** Bikram Datt Pant, Asdrubal Burgos, Pooja Pant, Alvaro Cuadros-Inostroza, Lothar Willmitzer, Wolf-Rüdiger Scheible

**Affiliations:** ^1^Max Planck Institute for Molecular Plant Physiology, D-14476 Potsdam-Golm, Germany; ^2^Plant Biology Division, The Samuel Roberts Noble Foundation, Ardmore, OK 73401, USA

**Keywords:** Lipidomics, lipid remodeling, microRNA399, phosphorus starvation, PHO2, PHR1, triacylglycerol.

## Abstract

Lipid remodeling is one of the most dramatic metabolic responses to phosphorus (P) starvation. It consists of the degradation of phospholipids to release the phosphate needed by the cell and the accumulation of glycolipids to replace phospholipids in the membranes. It is shown that PHR1, a well-described transcriptional regulator of P starvation of the MYB family, largely controls this response. Glycerolipid composition and the expression of most lipid-remodeling gene transcripts analysed were altered in the *phr1* mutant under phosphate starvation in comparison to wild-type plants. In addition to these results, the lipidomic characterization of wild-type plants showed two novel features of the lipid response to P starvation for *Arabidopsis*. Triacylglycerol (TAG) accumulates dramatically under P starvation (by as much as ~20-fold in shoots and ~13-fold in roots), a response known to occur in green algae but hardly known in plants. Surprisingly, there was an increase in phosphatidylglycerol (PG) in P-starved roots, a response that may be adaptive as it was suppressed in the *phr1* mutant.

## Highlight

This study reveals that the transcription factor PHR1 controls phospholipid/glycolipid substitution during P starvation, and that *Arabidopsis* accumulates triacylglycerol during P starvation. In roots this phenotype is also under control of PHR1.

## Introduction

Phosphorus (P) is an essential macronutrient for many vital processes but is often not easily available. The common form of P in soil and available for plants is inorganic phosphate (Pi). The abundance of Pi in soil is frequently low, as it is present in sparingly soluble minerals or bound to organic compounds. Plants thus have adapted to deal with P starvation in multiple ways. As a response to low Pi, plants can modify root growth and architecture (Lopez-Bucio *et al.*, 2002; Svistoonoff *et al.*, 2007), secrete organic acids to acidify the rooting medium and increase solubility of mineral phosphates (Raghothama, 1999), increase Pi uptake capacity (Mudge *et al.*, 2002), and trigger mechanisms to use P more efficiently (Denton *et al.*, 2007; Lambers *et al.*, 2012). One of these mechanisms is the activation of several branches of lipid metabolism that lead to major changes in lipid composition (Nakamura, 2013). This allows plants to exploit the Pi contained in phospholipids, where 15–30% of the organically bound Pi in the cell resides (Poirier *et al.*, 1991).

Lipid remodeling during P starvation consists essentially of the degradation of phospholipids to release Pi, and the synthesis of glycolipids to substitute phospholi pids. Sulphoquinovosyl diacylglycerol (SQDG) is synthesized to replace phosphatidylglycerol (PG) in the chloroplast (Essigmann *et al.*, 1998). Extraplastidial phospholipids are replaced by digalactosyl diacylglycerol (DGDG), restricted to the plastid under normal conditions, but found in extraplastidial membranes upon P starvation (Hartel *et al.*, 2000). At the molecular level, P starvation induces the expression of SQDG genes (*SQD1* and *2*) (Essigmann *et al.*, 1998; Yu *et al.*, 2002), the genes encoding the enzymes which synthesize the sulpholipid head group and add the head group to diacyl glycerol (DAG), respectively. Four galactosyltransferases are induced upon P starvation in Arabidopsis, leading to the accumulation of DGDG. Two of these enzymes [monogalactosyl diacylglycerol 2 and 3 (MGD2 and 3); Awai *et al.* (2001); Kobayashi *et al.* (2009)] synthesize MGDG. The other two enzymes (digalactosyl diacylglycerol 1 and 2) convert MGDG into DGDG. DGD1 is the housekeeping enzyme for DGDG synthesis (Dörmann *et al*., 1999), but the expression of its gene is nevertheless induced under P starvation (Kelly and Dörmann, 2002; Misson *et al.*, 2005). DGD2, on the other hand, is specifically induced under P starvation (Misson *et al.*, 2005; Morcuende *et al.*, 2007; Nakamura, 2013). The degradation of phospholipids occurs in at least three diffe rent ways. One possibility is that phospholipids are cleaved by non-specific phospholipases C4 and C5 (NPC4 and 5), relea sing the head group from DAG (Nakamura *et al.*, 2005; Li *et al.*, 2006; Gaude *et al.*, 2008). Alternatively, phospholipase D Z2 (PLDZ2) degrades phospholipids to phosphatidic acid (PA; Cruz-Ramirez *et al.*, 2006). The subsequent enzymatic step is the degradation of PA to DAG and is carried out by phosphatidate phosphohydrolases 1 and 2 (PAH1 and PAH2) (Nakamura *et al.*, 2009). In a third pathway, phosphol ipids are first degraded by lipid acyl hydrolases (LAHs) into fatty acids and glycerophosphodiester (GPD), which is later hydrolysed to glycerol-3-phosphate and an alcohol (Cheng *et al.*, 2011). Global gene expression microarray studies confirmed the up-regulation of the corresponding transcripts of all these genes in response to P starvation and also reported altered expression of yet more genes involved in lipid metabolism, for some of which their specific biological role in response to P starvation remains to be determined (Misson *et al.*, 2005; Morcuende *et al.*, 2007; Woo *et al.*, 2012).

Although P starvation-triggered lipid remodeling has been explored to a large extent, the regulation of lipid remodeling has remained elusive until now. The best known transcription factor regulating responses to P starvation is the MYB family factor PHR1 (Rubio *et al.*, 2001; Nilsson *et al.*, 2007). This transcription factor binds to P1BS elements with the consensus sequence ‘GNATATNC’ in the promoters of a large number of P starvation-responsive genes (Franco-Zorrilla *et al.*, 2004; Bustos *et al.*, 2010). In consequence, the loss-of-function mutant *phr1* grows more slowly than the wild type when exposed to P stress (Rubio *et al.*, 2001). The *phr1* mutant phenotype also includes a defective accumulation of anthocyanins, starch and sugar, an alteration in the shoot to root ratio, and impaired induction of multiple genes known to respond to P starvation (Rubio *et al.*, 2001; Bari *et al.*, 2006; Nilsson *et al.*, 2007; Bustos *et al.*, 2010). In fact, the mutation affects the expression of ~60% and ~20% of the genes induced (at least 2-fold) upon P starvation in shoots and roots, respectively (Bustos *et al.*, 2010). PHR1 does not only affect local responses; together with microRNA399 (miR399) and PHO2, an E2 ubiquitin-conjugase, PHR1 constitutes a systemic signalling pathway that communicates shoot Pi status to the root (Bari *et al.*, 2006; Pant *et al.*, 2008).

The link between the response of lipid metabolism and PHR1 was previously addressed in a work studying the role of NPC5 on galactolipid accumulation (Gaude *et al.*, 2008). Using thin-layer chromatography–gas chromatography (TLC-GC) of fatty acid methyl esters and RNA gel blo analysis, the authors did not observe a significant effect of a null allele of *PHR1* (SALK_067629) on DGDG synthesis during P limitation, and thus dismissed a role in lipid re modeling. Their results were surprising because a number of lipid-remodeling genes induced upon P starvation contain P1BS motifs in their promoters (Rubio *et al.*, 2001; Franco-Zorilla *et al*., 2004; Misson *et al.*, 2005; Muller *et al.*, 2007), many participating in either phospholipid degradation or glycolipid accumulation. In view of such facts, it was of interest to assess whether the responses of other lipid classes besides DGDG were dependent on PHR1. Thus, the effect of the *phr1* mutation on lipid composition was assessed with an ultra performance liquid chromatography–mass spectrometry (UPLC-MS) platform (Giavalisco *et al.*, 2011), and quantitative reverse transcription–PCR (qRT–PCR) was used to analyse the expression of lipid-remodeling genes in the *phr1-1* mutant originally described by Rubio *et al.* (2001), here simply referred to as *phr1*. It was found that the loss of PHR1 affects the P starvation-induced decrease of phospholipids, and the accumulation of MGDG and SQDG in shoots and roots, as well as that of DGDG, in contrast to the results of Gaude *et al.* (2008). Lipidomic characterization of P starvation also revealed that the storage lipid triacylglycerol (TAG) strongly accumulates in *Arabidopsis* leaves and roots in response to P starvation. TAG accumulation in response to P starvation is a well-known phenotype for green algae (Weers and Gulati, 1997; Lynn *et al.*, 2000; Gong *et al.*, 2013; Iwai *et al.*, 2014), but so far has only been reported in plants for cell cultures of black mustard (Fife *et al.*, 1990).

## Materials and methods

### Plant material and growth conditions

*Arabidopsis thaliana* seedlings were germinated and grown under constant light (~120 μmol photons m^–2^ s^–1^) at 22 °C in an axenic hydroponic system with either P-rich (3mM) or low P (30 μM) nutrient solution, using KH_2_PO_4_/K_2_HPO_4_ [potassium phosphate (KPi), pH 5.7] as the source of Pi. After 12 d, seedlings growing in P-rich conditions were supplied with fresh P-rich medium and seedlings growing in low P conditions were supplied with Pi-free medium and grown for another 4 d. The nutrient composition of the media was as described previously (Morcuende *et al.*, 2007), but no glutamine was added and NH_4_NO_3_ was increased to 2mM. Shoots and roots were dissected under ambient conditions, washed in deminera lized water, blotted on tissue paper, and then snap-frozen in liquid nitrogen before storage at –80 °C. Six replicates of *phr1* (*phr1-1*; Rubio *et al.*, 2001), *pho2* (Delhaize and Randall, 1995), miR399d overexpressers (Bari *et al.*, 2006), and Col-0 wild-type controls were obtained for each nutritional condition and organ.

### Sequence analysis and gene expression analysis by qRT–PCR

The motif ‘GNATATNC’ was searched in the full sequences of lipid-remodeling genes including the promoter sequence comprised within 1kb upstream of the 5′ untranslated region (UTR), using a standard text editor. RNA isolation, cDNA synthesis, and qRT–PCR expression quantification were performed as previously described (Czechowski *et al.*, 2005; Pant *et al.*, 2009). Total RNA isolated from roots and shoots using TRIzol reagent (Life Technologies) was subjected to DNase I treatment using the TURBO DNA-free Kit (Life Technologies) according to the manufacturer’s instructions. cDNA was synthesized using SuperScript III Reverse Transcriptase (Life Technologies) according to the manufacturer’s instructions and cDNA quality was tested. The expression analysis of lipid-remo deling genes was performed using a 7900HT Real-Time PCR System (Applied Biosystems). QRT-PCR primer sequences are given in Supplementary Table S1 (available at *JXB* online).

### Sudan Red staining for localization of triacylglycerol accumulation

Seedlings were stained with Sudan Red 7B (Brundett *et al*., 1991). The staining solution was prepared as follows: 50mg of Sudan Red 7B were dissolved in 25ml of polyethylene glycol (PEG)-400 and heated at 90 °C for 1h; an equal volume of 90% glycerol was added after cooling. *Arabidopsis* seedlings were soaked in the staining solution overnight and washed several times with distilled water. The seedlings were imaged using a Nikon SMZ 1500 stereo microscope.

### Lipid extraction

Aliquots of frozen, powdered shoots (~100mg) were prepared in 1.5ml Eppendorf tubes chilled in liquid N_2_. Each aliquot was then suspended in 300 μl of 100% methanol, followed by 15min shaking at 70 °C. Subsequently, 200 μl of CHCl_3_ were added, followed by 5min incubation at 37 °C and the addition of 400 μl of analytical grade water. The samples were subsequently vortexed and centrifuged for 5min at 14 000rpm. Finally, 320 μl of the organic phase were desiccated in a SpeedVac overnight. For roots, 40mg frozen aliquots were used and all extraction volumes were reduced to half.

### UPLC-MS measurement

UPLC separation of the lipid extract was performed using a Waters Acquity UPLC system (Waters, http://www.waters.com), with a C_8_ reversed-phase column (100 mm×2.1 mm×1.7 μm particles; Waters). The mobile phases were water (UPLC MS grade; BioSolve) with 1% 1M NH_4_Ac, 0.1% acetic acid (Buffer A), and acetonitrile:isopropanol (7:3 v/v, UPLC grade; BioSolve) containing 1% 1M NH_4_Ac and 0.1% acetic acid (Buffer B). A 2 μl sample was loaded per injection, and the gradient, run with a flow rate of 400 μl min^–1^, was: 1min 45% A, 3min linear gradient from 45% A to 25% A, 8min linear gradient from 25% A to 11% A, 3min linear gra dient from 11% A to 0% A (100% B). After washing the column for 3min with 0% A, the buffer is set back to 45% A and the column is re-equilibrated for 4min (22min total run time).

The mass spectra were acquired using an Exactive mass spectro meter (Thermo-Fisher, http://www.thermofisher.com). The spectra were recorded alternating between full-scan and all-ion-fragmentation scan modes, covering a mass range from 150 m/z to 1500 m/z. The resolution was 10 000, with 10 scans s^–1^, restricting the loading time to 100ms. The capillary voltage was 3kV with a sheath gas flow value of 60 and an auxiliary gas flow of 35 (values are in arbitrary units). The capillary temperature was 150 °C, whereas the drying gas in the heated electrospray source was 350 °C. The skimmer voltage was 25V, whereas that of the tube lens was 130V. The spectra were recorded from 1min to 17min of the UPLC gradients.

### Peak identification and quantification

GeneData software was used to pre-process the chromatogram raw files; that is, baseline correction, chemical noise subtraction, chromatogram alignment, and peak detection. Pre-processing parameters were set as previously described in Giavalisco *et al.* (2011). After pre-processing, a list of detected peaks (retention time and m/z pairs) and a matrix with their respective intensities for each sample were obtained.

A targeted search for the glycerolipid species of interest was carried out using the in-house developed R package *grms* (available upon request; A. Inostroza-Cuadros *et al.*, unpublished), based on the library compiled by Giavalisco *et al.* (2011). The software first performs a retention time (RT) correction of the output matrix based on previously identified markers with known RT. Then, the compounds are searched by comparing their specific m/z, expected adduct, and RTs within user-given ranges. A mass tolerance of 10 ppm and an RT deviation of between 0.05min and 0.2min were used to identify the lipid species. Further confirmation was achieved by manually inspecting the chromatograms with the software Xcalibur (Thermo-Fisher).

MSMS fragmentation data were used to determine TAG composition (Supplementary Table S2 available at *JXB* online). Automated determination of TAG composition was carried out by querying chromatogram intensities for TAG fragments with a known mass to the RTs of TAG species using the package *grms*. The hits were filtered by retaining only those hits with a difference of ±0.01min from the analysed TAG species. Manual inspection of fragment data was performed using Xcalibur software.

### Data analysis

Data normalization for lipid compounds detected by UPLC-MS was performed using R software (Ihaka and Gentleman, 1996; Warnes *et al.*, 2012) as follows. The coefficient of variation was first calculated from raw chromatogram intensities for each compound. Then, the intensities of the compounds with 50% lower variation, excluding TAGs, were used as the normalization factor for all the compounds in the data set. Isomers, defined as compounds with the same exact mass but slightly different RTs, were summed up into the same isobaric species. Statistical analyses were performed at the class level, the acyl carbon group level (compounds with the same number of acyl carbons), and the species level. For the class level, the content for every class was taken as the sum of species for the given class. Similarly, the species with the same number of acyl carbons were summed up to analyse the responses at the acyl carbon group level. Analysis of variance (ANOVA) and *t*-tests (*P*<0.05) were carried out at the three levels. Statistical analysis of qRT-PCR data (ANOVA and *t*-tests, *P*<0.05) were carried out using 40-∆C_T_ values (Morcuende *et al.*, 2007).

## Results and Discussion

### Revisiting lipidomics of P starvation

The response of lipid composition to P starvation has been addressed in a number of reports as summarized in the Introduction. A description of the lipid composition of our data on P-starved *Arabidopsis* is included here for two purposes: first, as a basis to describe the changes caused by the genetic backgrounds analysed; and, secondly, because the lipidomic characterization which was carried out revealed that DAG and TAG accumulate in addition to glycolipids during phosphate starvation.

*Arabidopsis* plants were grown for 16 d under either Pi-replete or Pi-limiting conditions. After this period, shoots and roots were harvested and analysed separately. Isobaric species (also known as apparent molecular species, compounds of the same class with the same number of acyl carbons and double bonds, hereafter referred to only as species) of the phospholipid classes phosphatidylcholine (PC), phosphatidylethanolamine (PE), phosphatidylserine (PS), and phosphatidylglycerol (PG), the glycolipid classes MGDG, DGDG, and SQDG, and the neutral lipid classes DAG and TAG were comprehensively quantified.

**Fig 1.**
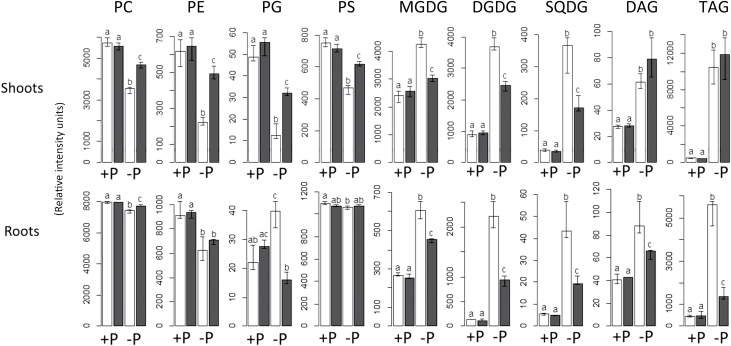
The change in lipid composition induced by P starvation is strongly influenced by the transcription factor PHR1. Values shown are the total content for each class in each condition. White bars represent wild-type plants and black bars represent *phr1* mutants. Units shown are relative and represent normalized intensities. The height of the bars indicates the median value. Error bars represent the interval between the first and the third quartiles. Letters (a–c) indicate statistical groups according to pairwise *t*-tests.

As expected, the levels of phospholipid classes (PC, PE, PS, and PG) decreased substantially during P starvation in shoots (Fig. 1). PG content, for instance, was only ~25% of that under P-replete conditions. On the other hand, phospholipids in the root did not decrease so strikingly. In fact, the only class decreasing significantly in the root upon P starvation was PE, while PG actually increased by ~80%.

The response of glycolipids was also noticeably different for both parts of the plant. DGDG increased upon phosphate starvation by 17-fold in roots, but only by 4-fold in shoots. MGDG increased by ~75% and ~130% in shoots and roots, respectively, whereas SQDG increased 10-fold in shoots and 8-fold in roots. Similar to the findings of a pre vious study (Misson *et al.*, 2005), the response of phospholipids in shoots was stronger than the response in roots. However, the response of glycolipids that was observed was noticeably stronger in the roots than in the shoots, as in Li *et al.* (2006). In the three cases, Misson *et al.* (2005), Li *et al.* (2006), and this study, the only three studies in which roots and shoots were analysed separately, the response of roots and shoots differ markedly.

**Fig. 2.**
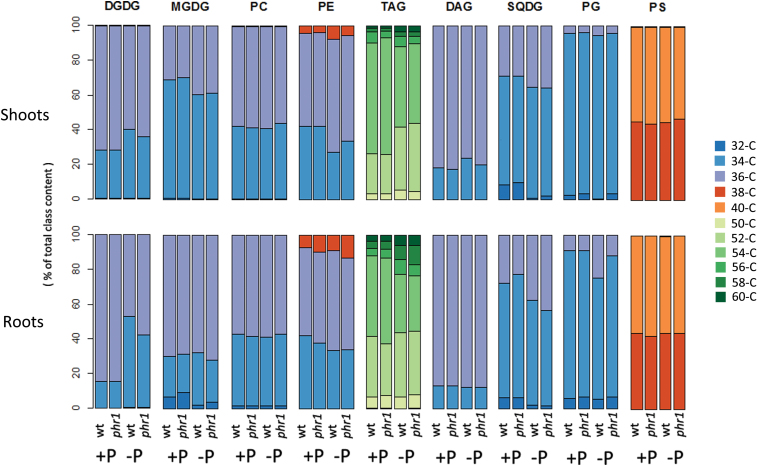
P starvation changes the composition of glycerolipid classes. Values show the percentage contribution of the different acyl groups (groups of lipid species with the same number of acyl carbons) to the total class content, the sum of the normalized intensities of all the compounds belonging to the same class.

Besides quantity, P status also influenced the composition of lipid classes (Fig. 2). Upon P starvation, the proportion of 36-C DGDG out of the total DGDG decreased at the expense of 34-C DGDG. At the species level, 36:5 and 36:6 DGDG account for practically all the increase in 36-C DGDG (Supplementary Fig. S1 at *JXB* online). In the root, both species together represented ~68% of total DGDG in P-replete conditions, but only ~21% under P starvation. This change occurred together with an increase in the proportion of 34:2 and 34:3 DGDG, which was much more marked in the root than in the shoot. Interestingly, in all other classes, the opposite occurred: the proportion of 34-C compounds decreased at the expense of 36-C compounds, both in shoots and in roots (Fig. 2). As with DGDG, this change in composition differed between shoots and roots. The proportion of 36-C compounds was at least 5% higher under P starvation in the shoot for PE and MGDG, and for PE, PG, and SQDG in the root.

The change in lipid species composition that occurs upon P starvation is probably a result of substrate specificities of the enzymes induced upon P starvation. The change of DGDG composition, first observed by Hartel *et al.* (2000), was suggested to be a result of P starvation-induced galactosidases (Kobayashi *et al.*, 2009). The enzymes MGD2 and MGD3 preferentially synthesize MGDG containing 16:0 and 18:2, and fewer 18:3 than MGD1 (Kobayashi *et al.*, 2009), while DGD2 uses preferentially 16:0 (Kelly *et al.*, 2003) in contrast to the housekeeping enzyme DGD1. The different substrate specificity results in an increased C16/C18 ratio in DGDG under P starvation (Kelly *et al.*, 2003). The more pronounced change in the C16/C18 ratio in roots than in shoots can also be attributed to these galactosidases, as the double mutation *mgd2mgd3* was reported to have a more marked effect on lipid composition in P-starved roots than in P-starved leaves (Kobayashi *et al.*, 2009).

It is possible that the role of the change in DGDG composition is to maintain the properties of extraplastidial membranes, as this is the final destination of the major portion of DGDG produced under P starvation (Hartel *et al*., 2001). The contents of 34-C species in PC and PE, the main extraplastidial lipids, are substantially higher than those in DGDG during normal conditions when it is restricted to the plastid. As mentioned before, in contrast to DGDG, other classes displayed the opposite trend, a decreased proportion of 34-C compounds at the expense of 36-C compounds. Further studies would be necessary to elucidate whether other enzymes are necessary for this change in the composition of other lipid classes, or if it can simply be explained as a consequence of the accumulation of 34-C DGDG and a possible decreased availability of 34-C DAG substrates.

The increase of PG in P-starved roots is a surprising finding, as all phospholipid classes would be expected to decrease upon P starvation. Although 34-C PG and 36-C PG both increased, the increase in 36-C PG was especially marked (Supplementary Fig. S1 at *JXB* online). Little is known about 36-C PG, although its presence in *Arabidopsis* and responsiveness to heat stress were reported previously (Burgos *et al.*, 2011). It is possible that its origin is extraplastidial as in other plant species. 36-C PG has been found in plasma membrane preparations (Lynch and Steponkus, 1987) and mitochondria (Dorne and Heinz, 1989). Furthermore, it is widely accepted that chloroplast PG is exclusively of plastidic origin, contai ning a maximum of 34 acyl carbons (Roughan, 1985).

The increase of PG that we observe in P-starved roots suggests that the complete spectrum of responses to P starvation is still unknown. Plants may face P shortage, limitation, or starvation at different points during their life cycle. The plants used in this study were grown for 12 d in a low-P medium, and 4 d in a medium without P before being harvested. The differences in phospholipid content observed for roots were mild in comparison with shoots or with what has been observed in other studies. It is thus likely that the seedlings faced a stronger limitation of P in the shoot than in the root. The low amount of P initially present in the medium probably was sufficient to allow almost normal levels of PC or PS in the root. In addition, the existing P may be sufficient to allow the increase observed in PG. Further work is needed to assess the physiological meaning of increased PG in P-starved roots and to determine whether this response occurs at later developmental stages.

### P starvation induces the accumulation of DAG and TAG

**Fig. 3.**
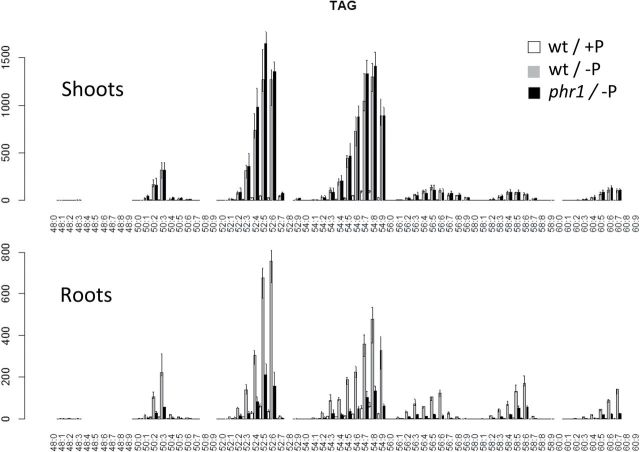
P starvation causes the accumulation of TAG in *Arabidopsis*. The factor PHR1 mediates this effect in roots but not in shoots according to UPLC-MS lipid profiling. Values shown are median relative intensities corresponding to wild-type plants growing at 3mM Pi (white bars) and 0mM Pi (grey bars), and *phr1* plants at 0mM Pi (black bars). Error bars represent the interval between the first and the third quartiles.

It is well known that plants store carbon as plastidic starch during P limitation (Ariovich and Cresswell, 1983; Fredeen *et al.*, 1988; De Groot *et al.*, 2001; Morcuende *et al.*, 2007). This is usually explained through reduced export of carbon, in the form of triosephosphates, from the chloroplasts by the triosephosphate–phosphate antiporter and by attenuation of allosteric inhibition of ADP-glucose pyrophosphorylase by Pi (Preiss, 1984; Stark *et al.*, 1992; Tiessen *et al.*, 2002). The finding that *Arabidopsis* also accumulated carbon as storage lipids (i.e. TAG) under P starvation (Figs 1 and 3) is molecularly unexplored. To the authors’ knowledge, this widely overlooked phenotype was only characterized to a certain extent in cell cultures of *Brassica nigra* (Fife *et al.*, 1990). Here it is reported that TAG content increased as much as ~20-fold in shoots and ~13-fold in roots upon P starvation. After applying the Sudan Red test to the P-starved seedlings, TAG accumulation could be observed to be greater in the cotyledons, although the staining in all the leaves was already substantially higher than in the P-replete seedlings (not shown).

As with DGDG, the TAG accumulating under P starvation has a different composition to that present in plants grown at high P. 52-C and 54-C TAG represented together >80% of total TAG under both conditions. However, the proportion of 52-C TAG of total TAG was ~17% and ~12% higher under P starvation in shoots and roots, respectively, at the expense of 54-C TAG (Figs 2 and 3). The other remarkable change in class composition was that of 34-C DGDG in roots, which more than doubled upon P starvation. Interestingly, 34-C DGDG and 52-C TAG both contain 16-C (Supplementary Table S2 at *JXB* online). As the content of 16-C chains increased for DGDG and TAG, it is possible that 16-C chains that are not allocated to DGDG are shuffled to TAG.

DAG, the other neutral lipid class analysed, increased by ~120% in both shoots and roots (Fig. 1). In the shoot, its composition changed slightly only during P starvation, with the proportion of 34-C DAG increasing at the expense of 36-C DAG (Fig. 2). The accumulation of DAG can occur for several reasons. DAG is a metabolic intermediate in the synthesis of glycolipids and most phospholipids (Browse and Somerville, 1991), and it can also be a product of phospholipid degradation (Nakamura *et al*., 2005, 2009). For DGDG accumulation during P starvation, DAG is an obligate intermediate. It would be a product either of NPC4/5 or of PAH1/2 and a substrate for MGD2/3. DAG is also a precursor of TAG (Zhang *et al.*, 2009). It is possible that the increase in DAG is a consequence of the accumulation of both DGDG and TAG. Remarkably, the three lipid classes contain a higher amount of 16-C chains upon P starvation.

### P starvation does not induce TAG assembly genes nor the fatty acid synthesis genes *PDH-E1a* and *BCCP2*

In *Arabidopsis*, TAG accumulation in leaves has been reported previously during leaf senescence (Kaup *et al.*, 2002), under cold stress (Degenkolbe *et al.*, 2012), and under nitrogen (N) limitation (Yang *et al.*, 2011). For these last two cases, it is possible that TAG accumulation is a way to store carbon in an inactive form, when photosynthesis is not as limiting as other factors important for plant growth. Although the mechanism for TAG accumulation in cold stress has not been explored, some information is available on its regulation du ring N stress and leaf senescence. Transcript and protein levels for diacylglycerol acyl transferase 1 (*DGAT1*, *At2g19450*), the enzyme that synthesizes TAG from DAG and acyl-CoA, increase with age and rise sharply during senescence (Kaup *et al.*, 2002). During N limitation, abscisic acid (ABA) regulates the accumulation of TAG via the induction of *DGAT1* by the transcription factor ABA insensitive 4 (*ABI4*, *At2g40220*) (Pourtau *et al.*, 2004). Interestingly, senescence can be induced by ABA and high availability of carbon relative to N (Pourtau *et al.*, 2004). These two pieces of evidence suggest that accumulation of TAG during N limitation and leaf senescence occurs through the same mechanism.

To explore how TAG accumulation is regulated during P starvation, the expression of both *DGAT1* and *ABI4* was investigated, but neither of the two gene transcripts was induced during P starvation (Supplementary Table S3 at *JXB* online). Previous studies have found that the responses to P starvation and ABA signalling are largely non-overlapping (Trull *et al.*, 1997; Franco-Zorrilla *et al.*, 2004), suggesting that TAG accumulation during P starvation is not mediated by ABA. The expression of several genes known for their role in seed oil accumulation was further analysed. Three of these genes are involved in TAG assembly: *PDAT1* (*At5g13640*; Zhang *et al.*, 2009), *ROD1* (*At3g15820*; Lu *et al.*, 2009), and *LPCAT2* (*At1g63050*; Xu *et al.*, 2012). Two more genes, *PDH-E1a* (*At1g01090*) and *BCCP2* (*At5g15530*), participate in fatty acid synthesis (Cernac *et al.*, 2006; To *et al.*, 2012). It was found that none of the five genes was up-regulated upon P starvation in wild-type plants (Supplementary Table S3). In agreement with this finding, no P1BS elements were found in the promoters of these genes when the ‘GNATATNC’ motif was searched for (not shown). Further work is thus needed to elucidate what part of metabolism is stimulated to accumulate TAG upon P starvation. As the enzymes are not regulated at the transcriptional level, it is possible that their basal levels are sufficient to catalyse the accumulation of TAG during P starvation. Further testing of *dgat1* and *pdat1* mutants will help to elucidate if this is the case and the respective contribution of the two enzymes to TAG accumulation.

### PHR1 regulates lipid-remodeling genes during P starvation

In order to investigate the importance of the transcription factor PHR1 in lipid remodeling under P starvation, P1BS motifs were searched for in a set of lipid metabolic genes either known to be involved in lipid remodeling or previously suggested to participate in the response of lipid metabolism to P starvation. The effect of PHR1 on the induction of this set of genes upon P starvation was also investigated by qRT–PCR using the knockout mutant *phr1-1* (Rubio *et al.*, 2001). The genes analysed were *NPC4* (*At5g20410*), *NPC5* (*At2g11810*), *PLDZ2* (*At3g05630*), *PAH1* (*At3g09560*), *PAH2* (*At5g42870*), *PLA2A* (*At2g26560*), *GDPD5* (*At1g74210*), and *GDPD6* (*At5g08030*), involved in phospholipid degradation, and *MGD2* (*At5g20410*), *MGD3* (*At2g11810*), *DGD1* (*At3g11670*), *DGD2* (*At4g00550*), *SQD1* (*At4g33030*), and *SQD2* (*At5g01220*), involved in glycolipid synthesis.

**Fig. 4.**
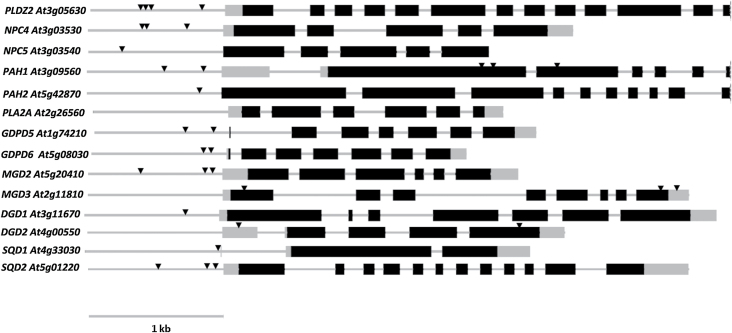
Genes involved in phospholipid degradation and glycolipid biosynthesis contain PHR1-binding sites (P1BS). Exons are shown as black boxes, the promoter and introns as a grey line, and UTRs as grey boxes. The position of P1BS sites in the promoters is indicated by triangles. The 1kb region upstream of the 5′ UTR was regarded as the promoter and was searched for P1BS elements together with the rest of the gene sequence.

The large majority of the genes tested contain P1BS motifs according to the sequence analysis performed (Fig. 4). The numbers of motifs vary greatly—*PLDZ2* contains four sites while *NPC5* and *PAH2* contains only one. Interestingly, *MGD3* and *DGD2*, well known to be responsive to phosphate starvation, contain no P1BS motifs in their promoters, but do in their UTRs and exons. Similarly, *PAH1* contains P1BS elements in the exons as well as in the promoter.

The expression of almost all of the genes mentioned above was up-regulated upon P starvation either in shoots or in roots, which is in agreement with microarray studies on P starvation (Misson *et al.*, 2005; Bustos *et al.*, 2010; Woo *et al.*, 2012). Interestingly, the magnitude of the induction was different for all of the genes, as was the influence of PHR1 in this induction, judging by the effect of the *phr1* mutation. There was not a linear correlation between the number of P1BS motifs, the magnitude of induction under phosphate starvation, and the influence of PHR1. For instance, in the shoot, *GDPD6* has a higher induction during P starvation than *PLDZ2.* Moreover, the effect of *phr1* on the induction was stronger in *GDPD6*. However, *PLDZ2* contained four P1BS motifs in the promoter, while *GDPD6* only contained two. Therefore, there is not a direct correlation between the number of P1BS sites and their induction by PHR1. The markedly different effect of the mutation in the expression of the two genes could suggest rather that, at least for some genes, the expression upon P starvation does not depend exclusively on PHR1, and additional regulatory factors could play a role in their induction.

**Fig. 5.**
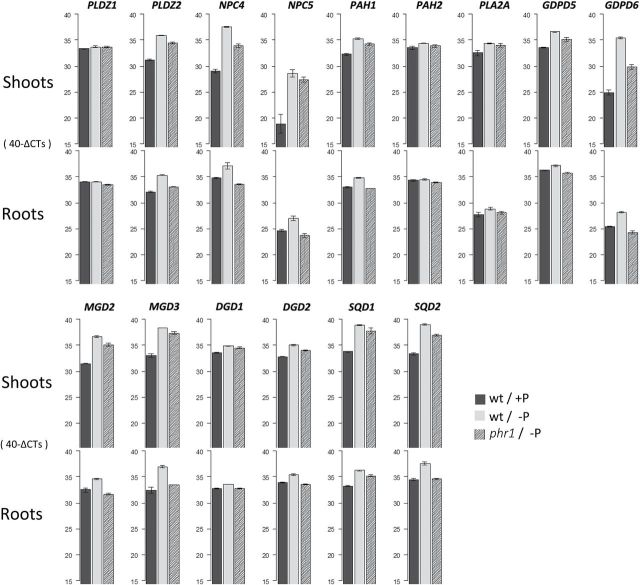
Expression of most lipid-remodeling genes during P starvation is under the control of PHR1. The influence of PHR1 appears stronger in the root according to qRT–PCR data. The values are expressed in 40-∆CTs, where ∆CT is the difference between the CT (threshold cycle number) of a tested gene and the reference gene (*UBQ10, AT4G05320*). The highest value possible is 40, as a qPCR run stops after completing 40 cycles. Values shown correspond to wild-type plants growing at 3mM Pi (black bars) and 0mM Pi (grey bars), and *phr1* plants at 0mM Pi (dashed bars). Bar heights represent the average of two technical and two biological replicates. Error bars depict one SD.

The response of the set of gene transcripts analyzed was different in shoots or roots (Fig. 5). In fact, the induction of practically all of them was stronger in the shoot than in the root. For instance, while *NPC4* had an ~8.5-fold induction in the shoot, it was induced only by ~2.4-fold in the root (Fig. 5; Supplementary Table S3 at *JXB* online). The differential behaviour in transcriptional programmes between shoots and roots was already noticed by Woo *et al.* (2012), who showed that different regulons operate upon P starvation in the two parts of the plant. Interestingly, the effect of the *phr1* mutation appeared to be more critical in the root even though these gene transcripts had a higher induction in the shoot. In the *phr1* shoot, the genes were induced by more or less half the wild-type response (Fig. 5, Supplementary Table S3 at *JXB* online), while in the root the induction of most genes was practically abolished. Only four gene transcripts (*PLDZ2*, *PLA2A*, *MGD3*, and *SQD1*) were still found to be slightly induced in *phr1* roots, none of them >2-fold Fig. 5, Supplementary Table S3 at *JXB* online.

### The change in lipid composition during P starvation is altered in the mutant phr1

Lipid profiling of *phr1* suggested strongly that PHR1 regulates lipid composition (Figs 1–3). While *phr1* showed a wild-type-like lipid composition in P-replete conditions, the profile was clearly different during P starvation (Figs 1, 2). In the *phr1* shoot, phospholipids did not decrease as strongly as in the wild-type shoot, whereas glycolipids did not accumulate to wild-type levels in any case. DAG and TAG increased somewhat more in *phr1* shoots than in wild-type shoots; however, no significant (*P*-value <0.05) difference was observed.

In the root, *phr1* had a mild effect on phospholipids, as only the total content of PC and PG was significantly different relative to the wild type, keeping in mind that in wild-type root phospholipids were already not very responsive to P starvation. Interestingly, the increase in PG reported for roots under P starvation is suppressed by the *phr1* mutation (Fig. 1; Supplementary Fig. S1 at *JXB* online), suggesting that the increase in PG is not a casual finding, but rather part of a transcriptional programme.

Unlike *phr1* shoots, TAG accumulation was severely affected in *phr1* roots. P-starved *phr1* roots had only ~25% of the TAG measured in P-starved wild-type roots. Similarly, the increase in DAG and glycolipid content was significantly affected. Whether *phr1* affected the change in the composition of lipid classes observed under P starvation was also analysed. The proportion of 36-C DGDG (relative to total DGDG) dropped by 37% in wild-type roots, but only by 27% in *phr1* roots (Fig. 2). Similarly, the proportion of 34-C PE dropped ~15% in wild-type shoots, but only ~8% in *phr1* shoots. In contrast, the proportion of 36-C SQDG increased only 8% in wild-type roots, but 17% in *phr1* roots. Altogether, the results indicate that PHR1 regulates lipid composition and remodeling during P starvation, and has potentially different roles in shoots and roots.

Whether of large or small magnitude, a PHR1-independent response was observed at the level of both gene expression and lipid composition. As mentioned, additional factors probably control lipid metabolism during P starvation. As shoots and roots were differentially affected by the mutation, either these factors are present in different amounts in both parts of the plant or different factors are at play in the shoot and root. One candidate which may also modulate lipid composition is the PHR1 homologue PHR1-like1 (PHL1). Bustos *et al.* (2010) assessed the functional redundancy of this gene with PHR1. Their microarray data showed that the induction of a number of genes involved in lipid metabolism (*MGD2*, *MGD3*, *SQD2*, *DGD2*, *NPC5*, and *PLDZ2*) is more severely affected in the *phr1phl1* double mutant than in *phr1*. Remarkably, the double mutation did not affect gene expression in shoots and roots equally. For instance, the induction of *NPC5* and *PLDZ2* was more strongly affected in roots of the double mutant than in *phr1*. (Bustos *et al.* 2010) Thus, other factors in addition to PHR1, such as PHL1, may be responsible for the differential response of lipid composition in shoots and roots.

Although other regulators of lipid remodeling remain to be discovered, the behaviour of other transcription factors controlling other responses to P starvation can shed some light on the regulation of lipid metabolism. The transcription factors MYB62, WRKY75, and ZAT6 (Devaiah *et al*., 2007*a*, *b*, 2009) are all induced upon P starvation but control P responses differently. Both MYB62 and WRKY75 induce Pi uptake but negatively regulate root branching (Devaiah *et al*., 2007*a*, 2009), while ZAT6 represses Pi uptake and positively regulates root branching (Devaiah *et al.*, 2007*b*). On the other hand, ZAT6 and MYB62 negatively regulate the expression of a set of P starvation-inducible genes (Devaiah *et al.*, 2007*b*, 2009), while WRKY75 induces their expression (Devaiah *et al.*, 2007*a*). The expression of these three factors was assessed and the three of them were found to be significantly affected by the *phr1* mutation. *MYB62* was up-regulated in the root, and *ZAT6* was down-regulated in the shoot, while *WRKY75* was up-regulated in both organs (Supplementary Table S3 at *JXB* online). It is possible that the expression of these factors is adjusted upon P starvation to compensate the lack of PHR1, although it is not known if they also affect lipid composition. Remarkably, ABI4, a factor tested here for its involvement in TAG accumulation upon P starvation, was up-regulated by P starvation in *phr1* shoots, but not in roots (Supplementary Table S3). Interestingly, TAG accumulation was severely affected in roots but not in shoots, so ABI4 could be replacing PHR1 function in TAG accumulation upon P starvation. Overall, the interaction of these or other factors, suppressing and activating different responses at the same time, probably shapes the lipid profile during P starvation.

It is conceivable that the transcriptional regulation during P stress is governed by more than a handful of factors. A global expression study following gene expression during the short, medium and long term—up to 15 d―found 80 transcriptional regulators to have their expression altered (Misson *et al.*, 2005). Among the 80 genes, other the study identified MYB trans cription factors, genes from the SCARECROW family, and AP2 domain and zinc-finger proteins (Misson *et al.*, 2005). Not all of these genes necessarily participate in the P-stress-specific response, but the number suggests that the response to P stress is the outcome of interactions among members of an intricate network of transcription factors. Further lipid profiling of mutants for other transcription factors will probably contribute to better understanding of the regulation of lipid remodeling during P stress.

### PHO2 repression does not trigger lipid remodeling

PHR1, together with miR399 and PHO2, constitutes a systemic signalling pathway that communicates shoot P status to the root. Six miR399 genes exist in *Arabidopsis*. Their transcripts are strongly induced by low P status in a PHR1-dependent manner (Fujii *et al.*, 2005; Bari *et al.*, 2006). *PHO2* is a target of miR399 repression (Aung *et al.*, 2006; Bari *et al.*, 2006), and represses itself the expression a set of P starvation-inducible (PSI) genes under P-sufficient conditions (Bari *et al.*, 2006). The *pho2* mutant and miR399 overexpresser (*miR399-OX*) show a range of indistinguishable visible, physiological, and molecular phenotypes (Aung *et al.*, 2006; Bari *et al.*, 2006; Pant *et al.*, 2009), such as the constitutive expression of PSI transcripts in P-replete conditions. The function of miR399 as a phloem-mobile long-distance signal that reports P status between organs was revealed when *miR399-OX* shoots were grafted to wild-type roots, and miR399 was able to suppress *PHO2* in the roots but not in the grafted shoots themselves (Pant *et al.*, 2008). In order to assess if miR399 and PHO2 are involved in the P starvation-dependent regulation of lipid metabolism, the expression of lipid-remodeling genes and lipid composition were analysed in the *pho2* null mutant and in *miR399d-OX*.

It was found that neither of these genotypes produced lipid phenotypes nearly as pronounced as that of *phr1* (Supplementary Fig. S2 at *JXB* online). However, in comparison with the wild type, both genotypes displayed several significant differences either in total class content or at the species level (Supplementary Figs S2, S3). In *miR399d-OX* P-replete shoots, several species of TAG accumulated, suggesting that at least part of the TAG phenotype is dependent on miR399 (Supplementary Fig. S4). In addition, both genotypes displayed lower levels of PC and PS and higher DGDG in P-starved roots. In P-replete shoots, slightly lower MGDG, DGDG, and SQDG together with higher PE were observed.

As *PHO2* is strongly repressed during P starvation, only P-replete conditions were analysed for gene expression in both *pho2* and *miR399-OX* lines. The changes in gene expression were not as extensive as in *phr1*, as happened with lipid composition. Nevertheless, *NPC5* and *PLA2A* were slightly up-regulated in the shoot of both genotypes, while *GDPD6*, *MGD2*, and *MGD3* were down-regulated in P-starved *miR399d-OX* roots (Supplementary Table S3 at *JXB* online).

The mild phenotypes of *pho2* and *miRNA399d-OX* in P-replete conditions, in both gene expression and lipid composition, showed that *PHO2* repression alone is not enough to trigger lipid remodeling, indicating that lipid composition is not part of the P starvation constitutive responses that these lines exhibit. A first interpretation would be that local Pi concentration and not systemic signalling is a determinant for lipid remodeling. However, a previous study dissecting local and systemic responses to P starvation highlighted several lipid metabolic genes as part of the systemic response (Thibaud *et al.*, 2010). By using a split-root system, the authors showed that the availability of Pi in half of the root system diminished the induction of P starvation genes, including *MGD2*, *MGD3*, *SQD1*, *SQD2*, *DGD2*, *PLDZ2*, *NPC4*, and *NPC5*, in the half of the root system exposed to P starvation. Furthermore, the *pho2* mutation restored the induction of *MGD3* and *SQD2* (Thibaud *et al.*, 2010). This implied that there is signalling not only from P-starved organs (Bari *et al.*, 2006), but also from P-replete organs that counteract the local P starvation signalling in a PHO2-mediated manner. One possibility is that locally low Pi concentrations induce P starvation signalling—propagated by PHR1—that PHO2 integrates with signals of Pi sufficiency. Further testing of this hypothesis could potentially help in unravelling the complex regulation of responses to P starvation.

In conclusion, here robust evidence was presented that the MYB-related transcription factor PHR1 exerts control over the majority of the changes in lipid metabolism that occur during P starvation, as it affects the content of most of the classes analysed as well as the expression of lipid-remodeling genes. The remnant response observed in the *phr1* mutant and the notably different responses in shoots and roots suggests that other regulators also contribute to the regulation of lipid remodeling. In addition, it is reported that *Arabidopsis*, a higher plant, accumulates carbon in the form of storage lipids (TAG) during P limitation. Further work is necessary to elucidate the underlying molecular mechanisms.

## Supplementary data

Supplementary data are available at *JXB* online.

Figure S1. Effect of P starvation on the relative abundance of glycerolipid species in shoots and roots of *Arabidopsis*.

Figure S2. Effect of *pho2* and *miR399d-OX* genotypes in lipid composition under P starvation.

Figure S3. Changes caused by *phr1, miR399d-OX*, and *pho2* genotypes at the species level.

Figure S4. The *miR399d-OX* genetic background has a significant effect on the abundance of a number of TAG species, although total TAG content is not significantly affected.

Table S1. Primer sequences used for real-time qPCR expression profiling.

Table S2. Fragmentation of 52-C and 54-C TAG species.

Table S3. Effect of P starvation and different genetic backgrounds (*phr1*, *pho2*, and *miR399d-OX*) on the expression of lipid-remodeling genes, transcription factors, and genes involved in TAG accumulation.

Supplementary Data
