# Supplementary material for: The transcription factor PHR1 regulates lipid remodeling and triacylglycerol accumulation in Arabidopsis thaliana during phosphorus starvation
Source: J Exp Bot. 2015 Feb 13;66(7):1907–18. doi: 10.1093/jxb/eru535 (PMC4378627; doi:10.1093/jxb/eru535)
Supplement: Supplementary Data [file supp_66_7_1907__index.html]

The transcription factor PHR1 regulates lipid remodeling and triacylglycerol accumulation in Arabidopsis thaliana during phosphorus starvation — The transcription factor PHR1 regulates lipid remodeling and triacylglycerol accumulation in Arabidopsis thaliana during phosphorus starvation — Supplementary Data 

# The transcription factor PHR1 regulates lipid remodeling and triacylglycerol accumulation in *Arabidopsis thaliana* during phosphorus starvation

## Supplementary Data

Data files

**Files in this Data Supplement:**

- Supplementary Data - Supplementary Data
