# Supplementary material for: The transcription factor PHR1 regulates lipid remodeling and triacylglycerol accumulation in Arabidopsis thaliana during phosphorus starvation
Source: J Exp Bot. 2015 Feb 13;66(7):1907–18. doi: 10.1093/jxb/eru535 (PMC4378627; doi:10.1093/jxb/eru535)
Supplement: Supplementary Data [file supp_eru535_jexbot132878_file001.pdf]

The transcription factor PHR1 regulates lipid remodeling and triacylglycerol accumulation in *Arabidopsis thaliana* during phosphorus starvation.

Bikram Datt Pant, Asdrubal Burgos, Pooja Pant, Alvaro Cuadros-Inostroza, Lothar Willmitzer, Wolf-Rüdiger Scheible

SUPPLEMENTARY DATA

Supplementary Figure S1. Effect of P starvation on the relative abundance of glycerolipid species in shoots and roots of *Arabidopsis*.

Values shown represent median relative intensities corresponding to shoots (A) and roots (B) of wild-type plants growing at 3mM Pi (white bars) and 0mM Pi (gray bars), and of *phr1* plants at 0mM Pi (black bars). Error bars represent the interval between the 1st and the 3rd quartiles.

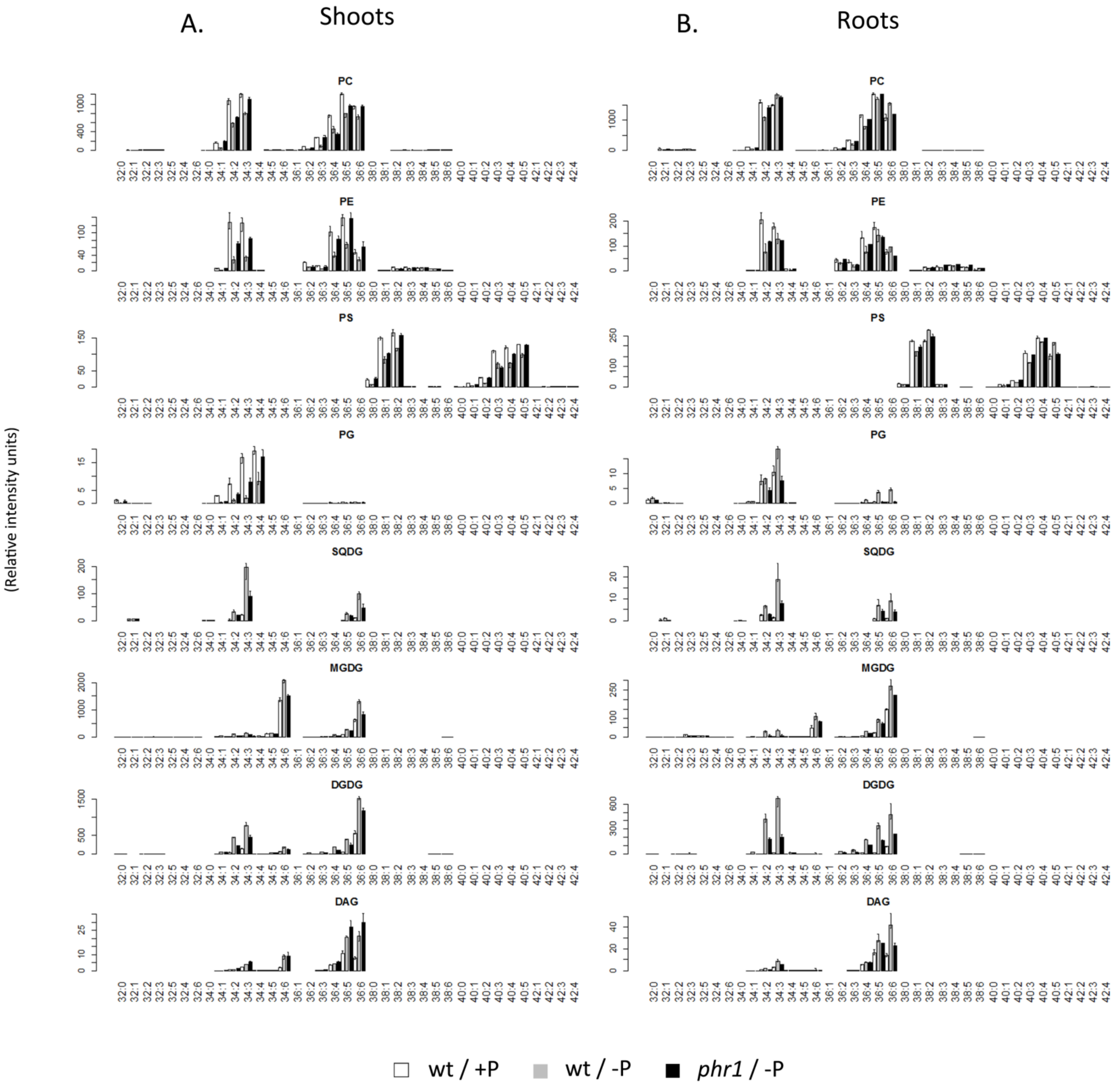

Supplementary Figure S2. Effect of *pho2* and *miR399d-OX* genotypes in lipid composition under P starvation.

The mutant *phr1* is included for comparison. Plotted values represent the sum of normalized intensities of all the species belonging to each class. Error bars represent the interval between the 1st and the 3rd quartiles. Statistical groups calculated with pairwise T-tests are indicated with letters (a-d). Color code: wild type (white), *pho2* (blue), *miR399-d OX* (red), and *phr1* (orange); 3mM Pi (+P, solid bars) and 0mM Pi (-P, dashed bars).

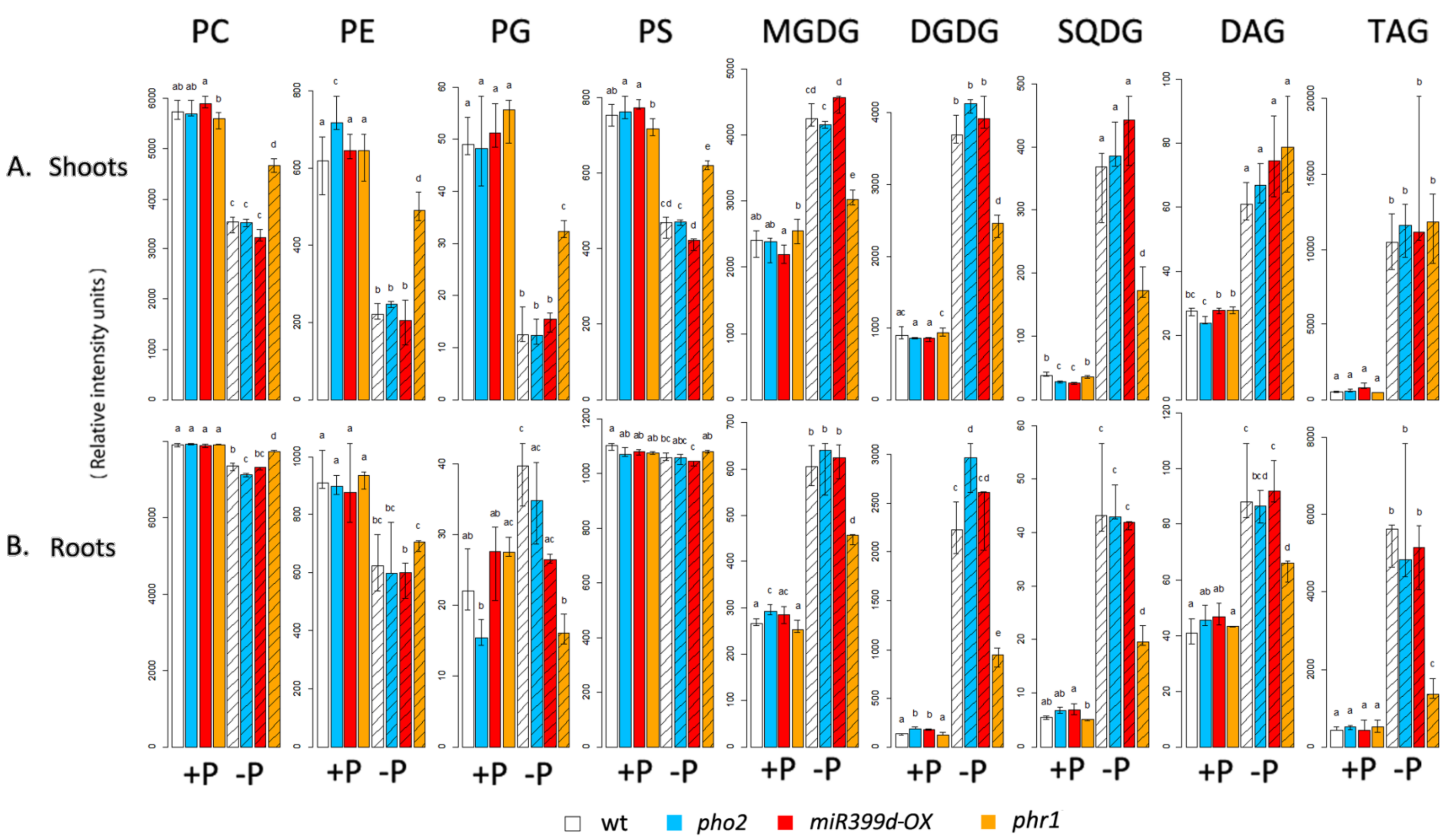

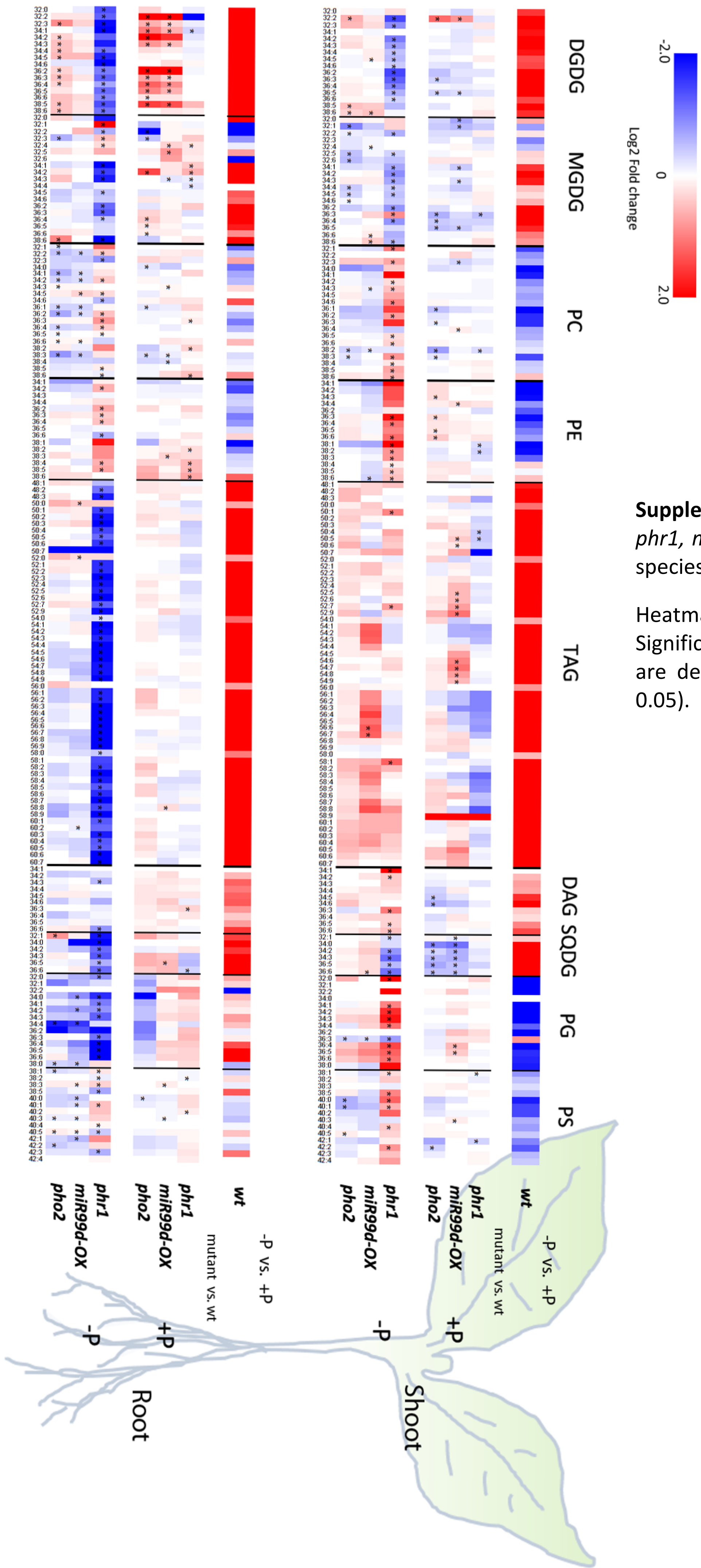

Supplementary Figure S3. Changes caused by *phr1, miR399d-OX* and *pho2* genotypes at the species level.

Heatmap colors indicate fold changes. Significant changes relative to the wild type are denoted with asterisks (T-test p-value < 0.05).

Supplementary Figure S4. The *miR399d-OX* genetic background has a significant effect on the abundance of a number of TAG species, although total TAG content is not significantly affected.

T-test p-values between wild type and *miR399d-OX* plants at 0mM Pi (-P) and 3mM Pi (+P) (**A**). Relative abundance of TAG species: wild type (gray), *miR399d-OX* (dark red) (**B**). Significant changes (p-value < 0.05) are highlighted with stars.

A.

wt *vs.* *miRNA399d OX*

	-P	+P
TAG 48:1	0.262	0.653
TAG 48:2	0.334	0.857
TAG 48:3	0.507	0.918
TAG 50:0	0.188	0.265
TAG 50:1	0.319	0.445
TAG 50:2	0.379	0.331
TAG 50:3	0.575	0.179
TAG 50:4	0.445	0.072
TAG 50:5	0.513	* 0.039
TAG 50:6	0.580	* 0.046
TAG 50:7	0.253	0.055
TAG 52:0	0.456	0.103
TAG 52:1	0.200	0.427
TAG 52:2	0.195	0.424
TAG 52:3	0.299	0.296
TAG 52:4	0.399	0.095
TAG 52:5	0.517	* 0.044
TAG 52:6	0.538	0.025
TAG 52:7	0.398	* 0.019
TAG 52:9	0.642	* 0.001
TAG 54:0	0.192	0.077
TAG 54:1	0.077	0.447
TAG 54:2	0.075	0.442
TAG 54:3	0.065	0.429
TAG 54:4	0.110	0.386
TAG 54:5	0.226	0.142
TAG 54:6	0.348	* 0.021
TAG 54:7	0.352	* 0.005
TAG 54:8	0.510	* 0.006
TAG 54:9	0.569	* 0.018
TAG 56:0	0.520	0.821
TAG 56:1	0.130	0.415
TAG 56:2	0.126	0.413
TAG 56:3	0.071	0.431
TAG 56:4	0.057	0.430
TAG 56:5	0.061	0.420
TAG 56:6	* 0.049	0.447
TAG 56:7	* 0.047	0.461
TAG 56:8	0.179	0.331
TAG 56:9	0.290	0.309
TAG 58:0	0.859	0.057
TAG 58:1	0.271	0.323
TAG 58:2	0.169	0.417
TAG 58:3	0.098	0.434
TAG 58:4	0.088	0.454
TAG 58:5	0.209	0.473
TAG 58:6	0.221	0.476
TAG 58:7	0.052	0.572
TAG 58:8	0.062	0.612
TAG 58:9	0.151	0.801
TAG 60:1	0.250	0.380
TAG 60:2	0.224	0.372
TAG 60:3	0.170	0.434
TAG 60:4	0.133	0.438
TAG 60:5	0.216	0.451
TAG 60:6	0.344	0.214
TAG 60:7	0.345	0.095

B.

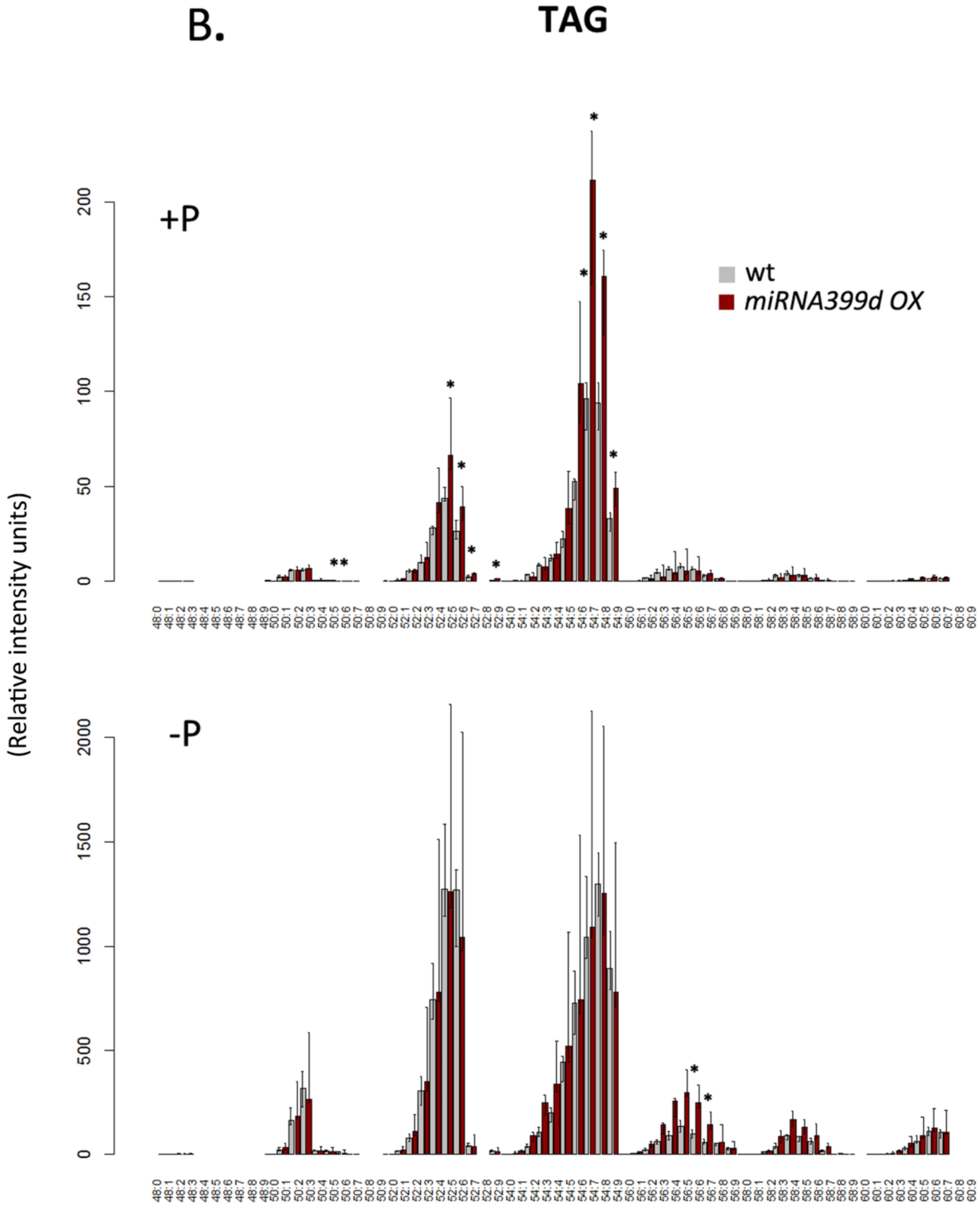

Supplementary Table S1. Fragmentation of 52-C and 54-C TAG species.

With the aim of exploring the composition of the most abundant TAG acyl groups, the masses of TAG fragments of 30 to 46 acyl carbons, with 0 to 6 unsaturations, were queried to TAG species MSMS spectra. The hits shown in the table had a maximum retention time (RT) difference of ± 0.01 min to the TAG species fragmented. For 52-C species there were only 34-C and 36-C fragments, implying 18-C and 16-C losses. It is well known that the bulk of PC and DAG, TAG precursor classes, contains either 34 or 36 acyl carbons, with a composition of 18-C + 16-C and 18-C + 18-C fatty acids, respectively (Browse and Somerville 1991). As only 16-C or 18-C fatty acid losses could be detected, the composition of 52-C TAG can be deduced as 18-C + 18-C + 16-C.

TAG species	formula	m/z (M + NH4)	RT	TAG fragment	formula	m/z (M + H)	RT	Predicted loss
52:0	C55H106O6	880.8347	15.8050	34:0	C37H70O4	579.5324	15.7979	18:0
52:1	C55H104O6	878.8105	15.3403	34:0	C37H70O4	579.5327	15.3352	18:1
52:1	C55H104O6	878.8105	15.3403	34:1	C37H68O4	577.5188	15.3371	18:0
52:2	C55H102O6	876.8018	15.3429	34:0	C37H70O4	579.5327	15.3352	18:2
52:2	C55H102O6	876.8018	15.3429	34:1	C37H68O4	577.5188	15.3371	18:1
52:2	C55H102O6	876.8018	15.3429	34:2	C37H66O4	575.5029	15.3440	18:0
52:3	C55H100O6	874.7854	15.0890	34:0	C37H70O4	579.5327	15.0858	18:3
52:3	C55H100O6	874.7854	15.0890	34:1	C37H68O4	577.5179	15.0816	18:2
52:3	C55H100O6	874.7854	15.0890	34:3	C37H64O4	573.4876	15.0909	18:0
52:3	C55H100O6	874.7854	15.0890	36:3	C39H68O4	601.5188	15.0986	16:0
52:4	C55H98O6	872.7701	14.8094	34:1	C37H68O4	577.5174	14.8149	18:3
52:4	C55H98O6	872.7701	14.8094	34:2	C37H66O4	575.5028	14.8036	18:2
52:5	C55H96O6	870.7544	14.5049	34:2	C37H66O4	575.5029	14.5042	18:3
52:5	C55H96O6	870.7544	14.5049	34:3	C37H64O4	573.4875	14.5038	18:2
52:6	C55H94O6	868.7390	14.1696	34:3	C37H64O4	573.4875	14.1696	18:3
52:6	C55H94O6	868.7390	14.1696	34:4	C37H62O4	571.4718	14.1700	18:2
52:7	C55H92O6	866.7237	13.7994	34:4	C37H62O4	571.4721	13.7918	18:3
52:7	C55H92O6	866.7237	13.7994	34:5	C37H60O4	569.4573	13.8007	18:2
52:9	C55H88O6	862.6936	12.8072	34:6	C37H58O4	567.4408	12.8145	18:3
52:9	C55H88O6	862.6936	12.8072	36:6	C39H62O4	595.4718	12.8154	16:3
54:1	C57H108O6	906.8403	15.5859	36:0	C39H74O4	607.5652	15.5873	18:1
54:1	C57H108O6	906.8403	15.5859	36:1	C39H72O4	605.5495	15.5824	18:0
54:2	C57H106O6	904.8335	15.5894	34:2	C37H66O4	575.5022	15.5983	20:0
54:2	C57H106O6	904.8335	15.5894	36:0	C39H74O4	607.5652	15.5873	18:2
54:2	C57H106O6	904.8335	15.5894	36:1	C39H72O4	605.5495	15.5824	18:1
54:2	C57H106O6	904.8335	15.5894	36:2	C39H70O4	603.5342	15.5890	18:0
54:3	C57H104O6	902.8173	15.3651	34:3	C37H64O4	573.4876	15.3732	20:0
54:3	C57H104O6	902.8173	15.3651	36:0	C39H74O4	607.5650	15.3635	18:3
54:3	C57H104O6	902.8173	15.3651	36:1	C39H72O4	605.5485	15.3561	18:2
54:3	C57H104O6	902.8173	15.3651	36:2	C39H70O4	603.5345	15.3589	18:1
54:3	C57H104O6	902.8173	15.3651	36:3	C39H68O4	601.5185	15.3624	18:0
54:4	C57H102O6	900.8012	15.1174	36:1	C39H72O4	605.5488	15.1106	18:3
54:4	C57H102O6	900.8012	15.1174	36:2	C39H70O4	603.5334	15.1104	18:2
54:4	C57H102O6	900.8012	15.1174	36:4	C39H66O4	599.5030	15.1192	18:0
54:5	C57H100O6	898.7859	14.8508	36:2	C39H70O4	603.5334	14.8536	18:3
54:5	C57H100O6	898.7859	14.8508	36:3	C39H68O4	601.5176	14.8441	18:2
54:5	C57H100O6	898.7859	14.8508	36:5	C39H64O4	597.4879	14.8553	18:0
54:6	C57H98O6	896.7697	14.5520	36:3	C39H68O4	601.5182	14.5559	18:3
54:6	C57H98O6	896.7697	14.5520	36:6	C39H62O4	595.4717	14.5567	18:0
54:7	C57H96O6	894.7540	14.2153	36:4	C39H66O4	599.5028	14.2177	18:3
54:7	C57H96O6	894.7540	14.2153	36:5	C39H64O4	597.4872	14.2097	18:2
54:8	C57H94O6	892.7389	13.8402	36:5	C39H64O4	597.4873	13.8433	18:3
54:8	C57H94O6	892.7389	13.8402	36:6	C39H62O4	595.4719	13.8439	18:2
54:9	C57H92O6	890.7234	13.4045	36:6	C39H62O4	595.4721	13.4048	18:3

Supplementary Table S2. Effect of P starvation and different genetic backgrounds (*phr1*, *pho2* and *miR399d-OX*) on the expression of lipid remodeling genes, transcription factors and genes involved in TAG accumulation.

Values are log2-transformed fold changes between two conditions/genotypes. These ratios were calculated from average expression levels (two technical and two biological replicates) of 40-ΔCTs. Significant changes (T-test p-value < 0.05) are denoted with stars.

-5.0 0 5.0
Log2 Fold change

			Shoots				Roots			
			wt -P vs. wt +P	*phr1* -P vs. wt -P	*pho2* +P vs. wt +P	*miR399d-OX* vs. wt +P	wt -P vs. wt +P	*phr1* -P vs. wt -P	*pho2* +P vs. wt +P	*miR399d-OX* vs. wt +P
Lipid remodeling genes	***PLDZ1***	*AT3G16785*	0.41	-0.02	0.38	0.14	0.01	* -0.63	0.07	-0.11
	PLDZ2	*AT3G05630*	* 4.69	* -1.45	-0.05	0.18	* 3.15	* -2.22	0.40	0.17
	NPC4	*AT3G03530*	* 8.47	* -3.56	* 0.51	-0.03	* 2.37	* -3.55	0.14	-0.05
	NPC5	*AT3G03540*	* 9.73	* -1.18	* 1.18	* 1.89	* 2.34	* -3.32	0.12	* 0.65
	PAH1	*AT3G09560*	* 3.01	* -1.04	-0.03	0.01	* 1.86	* -2.05	0.15	-0.23
	PAH2	*AT5G42870*	* 0.89	* -0.54	0.17	0.18	0.10	* -0.55	-0.10	-0.20
	PLA2A	*AT2G26560*	* 1.87	-0.35	* 0.89	* 1.68	* 1.21	* -0.76	-0.05	* 0.90
	GDPD5	*AT1G74210*	* 2.98	* -1.43	-0.10	0.29	* 0.92	* -1.41	0.28	-0.34
	GDPD6	*AT5G08030*	* 10.53	* -5.57	0.19	-0.11	* 2.76	* -3.88	-0.08	* -0.53
	MGD2	*AT5G20410*	* 5.20	* -1.59	0.06	-0.49	* 2.06	* -2.89	0.44	* -1.03
	MGD3	*AT2G11810*	* 5.35	* -1.04	-0.20	* -1.30	* 4.50	* -3.50	* 1.07	* -0.60
	DGD1	*AT3G11670*	* 1.32	-0.37	-0.04	-0.21	* 0.79	* -0.81	0.11	-0.28
	DGD2	*AT4G00550*	* 2.23	* -1.02	0.10	0.01	* 1.50	* -1.86	0.16	-0.39
	SQD1	*AT4G33030*	* 5.16	* -1.17	-0.12	-0.20	* 3.00	* -1.06	* 0.75	0.35
	SQD2	*AT5G01220*	* 5.65	* -2.10	-0.10	-0.05	* 3.11	* -2.95	0.35	-0.26
Genes involved in TAG accumulation	***DGAT1***	*AT2G19450*	0.36	0.33	-0.15	0.00	-0.13	* -0.69	-0.10	-0.35
	PDAT1	*AT5G13640*	0.50	0.17	-0.02	-0.16	-0.20	* -0.54	0.05	-0.49
	ROD1	*AT3G15820*	0.22	-0.04	-0.16	0.08	-0.35	* -0.69	0.02	* -0.70
	LPCAT2	*AT1G63050*	* -0.54	* 0.97	-0.27	0.06	-0.41	* -0.58	0.09	-0.44
	BCCP2	*AT5G15530*	* -0.90	* 0.54	-0.20	* -0.63	-0.37	* -1.59	0.26	* -0.62
	PDH-E1α	*AT1G01090*	-0.40	0.21	0.01	-0.19	* -1.02	-0.46	-0.16	* -0.75
Transcription factors	***ABI4***	*AT2G40220*	* -1.92	* 5.70	* 0.73	* -0.56	* -4.49	-0.48	* -3.97	* -4.52
	MYB62	*AT1G68320*	* 2.70	-0.13	0.40	0.34	0.16	* 2.68	* -1.20	* -0.86
	WRKY75	*AT5G13080*	-0.31	* 1.46	-0.40	* 1.06	* 1.61	* 0.88	-0.11	0.41
	ZAT6	*AT5G04340*	* -0.80	* -1.69	* -1.12	* -1.70	-0.25	0.08	* -1.37	* -0.79
	UBQ10	*AT4G05320*	0.00	0.00	0.00	0.00	0.00	0.00	0.00	0.00
